# Improving the use of research evidence in guideline development: 10. Integrating values and consumer involvement

**DOI:** 10.1186/1478-4505-4-22

**Published:** 2006-12-05

**Authors:** Holger J Schünemann, Atle Fretheim, Andrew D Oxman

**Affiliations:** 1 INFORMA/CLARITY Research Group, S.C. Epidemiologia, Istitituto Regina Elena, Via Elio Chianesi 53, 00144 Rome, Italy; 2 Norwegian Knowledge Centre for the Health Services, P.O. Box 7004, St. Olavs plass, N-0130 Oslo, Norway

## Abstract

**Background:**

The World Health Organization (WHO), like many other organisations around the world, has recognised the need to use more rigorous processes to ensure that health care recommendations are informed by the best available research evidence. This is the 10^th ^of a series of 16 reviews that have been prepared as background for advice from the WHO Advisory Committee on Health Research to WHO on how to achieve this.

**Objectives:**

We reviewed the literature on integrating values and consumers in guideline development.

**Methods:**

We searched PubMed and three databases of methodological studies for existing systematic reviews and relevant methodological research. We reviewed the titles of all citations and retrieved abstracts and full text articles if the citations appeared relevant to the topic. We checked the reference lists of articles relevant to the questions and used snowballing as a technique to obtain additional information. We did not conduct a full systematic review ourselves. Our conclusions based on the available evidence, consideration of what WHO and other organisations are doing and logical arguments.

**Key questions and answers:**

We did not find a systematic review of methods for integrating values in guidelines, but we found several systematic reviews that dealt with related topics.

Whose values should WHO use when making recommendations?

• Values, the relative importance or worth of a state or consequences of a decision (outcomes relating to benefits, harms, burden and costs), play a role in every recommendation. Ethical considerations, concepts that determine what is right, also play a role.

• The values used in making recommendations should reflect those of the people affected. Judgements should be explicit and should be informed by input from those affected (including citizens, patients, clinicians and policy makers).

• When differences in values may lead to different decisions or there is uncertainty about values, this should also be explicit. If differences in values are likely to affect a decision, such that people in different setting would likely make different choices about interventions or actions based on differences in their values, global recommendations should be explicit in terms of which values were applied and allow for adaptation after incorporating local values.

How should WHO ensure that appropriate values are integrated in recommendations?

• All WHO guideline groups should uniformly apply explicit, transparent and clearly described methods for integrating values.

• WHO should consider involving relevant stakeholders if this is feasible and efficient.

• WHO should develop a checklist for guidelines panels to help them to ensure that ethical considerations relevant to recommendations are addressed explicitly and transparently.

How should users and consumers be involved in generating recommendations?

• Including consumers in groups that are making global recommendations presents major challenges with respect to the impossibility of including a representative spectrum of consumers from a variety of cultures and settings. Nonetheless, consideration should be given to including consumers in groups who are able to challenge assumptions that are made about the values used for making recommendations, rather than represent the values of consumers around the world.

• WHO should establish a network to facilitate involvement of users.

• Draft recommendations should be reviewed by consumers, who should be asked explicitly to consider the values that were used.

How should values be presented in recommendations?

• Recommendations should include a description of how decisions were made about the relative importance of the consequences (benefits, harms and costs) of a decision.

• Values that influence recommendations should be reported along with the research evidence underlying recommendations.

• When differences in values would lead to different decisions or there is important uncertainty about values that are critical to a decision, this should be flagged and reflected in the strength of the recommendation.

• Adaptable guideline templates that allow for integration of different values should be developed and used when differences in values are likely to be critical to a decision.

## Background

The World Health Organization (WHO), like many other organisations around the world, has recognised the need to use more rigorous processes to ensure that health care recommendations are informed by the best available research evidence. This is the 10^th ^of a series of 16 reviews that have been prepared as background for advice from the WHO Advisory Committee on Health Research to WHO on how to achieve this.

Utilities, health state preferences or values are the desirability or preference that individuals exhibit for a particular health state [1]. Individuals usually assign less value to and have less preference for more impaired health states (e.g. death or dependency after a stroke) compared to other health states (e.g. full health or having a very mild stroke without serious sequela). In this document we will use the terms "values" to refer to the relative worth or importance of a health state or consequences (benefits, harms and costs) of a decision. It is primarily this concept of values that we focus on here.

Ethical or moral values also play a role in making health care recommendations [2-4]. These refer to concepts of what is right based on philosophical, humanistic or religious considerations. Ethical values can vary among individuals within a society and across societies or culture, and may influence recommendations and the implementation of recommendations. We will refer to these as ethical considerations.

Several formal methods exist to measure values in healthcare [5,6]. The principle methods are based on direct utility instruments including the standard gamble, time-trade off and visual analogue scales [5,6]. Direct preference-based instruments generate a value score for respondents' current health state or hypothetical states, typically on a 0.0 to 1.0 scale where 0.0 indicates dead and 1.0 indicates full health. Indirect methods offer alternatives to direct assessments and include multi-attribute utility tools and transformations based on quality of life assessments [7-10]. Multi-attribute utility tools ask respondents to describe their health state, and the value of that health state is calculated using a developed formula representing preferences of the general population. The Euroquol, Quality of Well-Being Index, and the Health Utilities Index are examples of this approach [9-11]. Transformations from generic health related quality of life tools utilize modelling techniques that transform quality of life scores into values [8]. Ranking scales and qualitative methods compliment these methods [6]. However, the application of their results is complicated by the fact that the reproducibility between the different methods is poor and each of the methods has strength and limitations.

Values play a role in every recommendation, either explicitly or implicitly. For instance patients who suffered an idiopathic deep venous thrombosis (DVT) usually receive treatment with adjusted dose warfarin for one year to prevent recurrent DVT and pulmonary embolism [12]. Continuing on standard-intensity warfarin beyond the treatment of one year will reduce his absolute risk for recurrent DVT by more than 7% per year for several years [13]. The burdens of treatment include taking a warfarin pill daily, keeping dietary intake of vitamin K constant, monitoring the intensity of anticoagulation with blood tests, and living with the increased risk of both minor and major bleeding. Patients who are very averse to a recurrent DVT would consider the benefits of avoiding DVT worth the downsides of taking warfarin. Other patients are likely to consider the benefit not worth the harms and burden. Another example refers to the different values patients with atrial fibrillation and their clinicians place on the adverse consequences of stroke and gastrointestinal bleed [14]. A third example relates to a health care recommendation about the combination of chemotherapy and radiotherapy versus radiotherapy alone in unresectable, locally advanced non-small cell lung cancer [15,16]. Compared with radiotherapy alone, the combination of chemotherapy and radiotherapy reduces the risk for death corresponding to a mean gain in life expectancy of a few months [15], but increases harm and burden related to chemotherapy. Thus, considering the values and preferences patients would place on the small survival benefit in view of the harms and burdens, guideline panels may offer a weak recommendation despite the high quality of the available evidence. Generally, there is agreement that the values that are used for comparing the relative benefits and downsides of interventions should be explicit [17-20].

In this paper we addressed the following questions:

• Whose values should WHO use when making recommendations?

• How should WHO ensure that appropriate values are integrated in recommendations?

• How should users and consumers be involved in generating recommendations?

• How should values be presented in recommendations?

Questions related to identifying important outcomes, group composition and equity considerations are addressed in other papers in this series [21-23].

## What WHO is doing now

The Guidelines for WHO Guidelines suggests that end users, and patients specifically, should be represented on guideline panels [24]. However, review of selected WHO guidelines did not yield information about the inclusion of end users or patients in guideline groups or the use of other methods to ensure appropriate integration of values and consumer involvement. The fact that WHO makes global recommendations presents challenges for obtaining appropriate representation of values, because values may differ across different cultures and settings.

## What other organisations are doing?

A number of guideline developers have invited individuals who could represent and understand the perspectives of stakeholders, including consumers [25]. However, in a recent survey of organizations and specialty societies that develop guidelines only approximately 25% regularly involved consumers in the process [26]. In another survey of 18 prominent organizations that develop guidelines approximately 50% regularly involved patients [27].

The UK National Institute for Health and Clinical Excellence (NICE) has adopted a very comprehensive approach to involving patients and consumers and has formed a patient involvement unit aiming to involve patients and carers in the development of individual clinical guidelines [20,28]. NICE consumer involvement can be categorized into four broad areas:

1) Stakeholder consultation

Organisations can register and comment at any stage during the development process. They can nominate patient groups and participate and comment on the development of a guideline at every stage from the suggestion of guideline topics, drafting of scopes, development and initial drafting of guidelines, to the second consultation draft.

2) Direct input

NICE committees and working groups are expected to include at least two members who play a crucial role by providing a patient/carer perspective to their discussions and decisions. They may be patients, carers or patient advocates. Vacancies are publicised via national patient and carer organisations, on a website or via the national press.

3) Indirect input

Examples include focus groups with patients, patient written testimonials and video-taped interviews with patients that were presented to a technology appraisal committee.

4) Dissemination of NICE guidance to and by patients

All NICE guidance is produced in versions written for patients, carers and the public. Copies can be downloaded from the NICE website or printed copies can be obtained by telephoning the NHS Response line. Patient organisations play an increasingly significant role in helping NICE disseminate its guidance to individual patients and carers and providing feedback.

The Scottish Intercollegiate Guidelines Network (SIGN) involves patients and patient representatives in the guideline development process. For each guideline, SIGN aims to search for evidence about patient important outcomes after having identified a guideline topic, but this is not done consistently (R. Habour, personal communication). SIGN also aims to include patient representatives and guideline users in the process at all stages during the development process [17]. The UK National Health System (NHS) Health Technology Assessment program also systematically involves public advocates in their work [29-32]. Various speciality societies describe that representation of consumers on guideline panels (at least one member) is required which is often described as representation by a patient advocacy group [33].

The Cochrane Collaboration, which produces systematic reviews, but does not make recommendations, has made consumer involvement an integral part of its work of producing and disseminating systematic reviews and demonstrated the feasibility of international consumer involvement projects. In 1998 approximately two thirds of Cochrane Collaboration review groups had consumer involvement [34]. There is also a Cochrane Consumers Network [35], which helps to provide consumer input into developing Cochrane reviews.

## Methods

The methods used to prepare this review are described in the introduction to this series [36]. Briefly, the key questions addressed in this paper were vetted amongst the authors and the ACHR Subcommittee on the Use of Research Evidence (SURE). We reviewed existing guidelines for guidelines to identify processes for integrating consumer values and consumer involvement. We based the current summary and recommendation on the work of prominent developers of guidelines. We also searched PubMed using "consumer" and "involvement" as search terms (MESH headings/keywords) for systematic reviews. We searched PubMed for systematic reviews on how guideline groups integrate values and preferences using the terms "guideline" and "values OR preferences OR utilities" (we identified 694 citations labelled as systematic reviews). We also searched the Cochrane library, Methodology registry and database using the keywords "guideline" and ("values" or "preferences"). We searched databases maintained by the Agency for Healthcare Research and Quality (AHRQ, [37]) and the Guidelines International Network (GIN, [38]), and reviewed information obtained from various organizations and our own files. We did not conduct a full systematic review. The answers to the questions are our conclusions based on the available evidence, consideration of what WHO and other organisations are doing and logical arguments.

## Findings

We did not find a systematic review of methods for integrating values in guidelines, but we found one systematic review that compared whether values differ between the general population and patients [39]. Another systematic review that focused on interventions to promote consumer involvement in developing healthcare policy and clinical practice guidelines did not find any comparative studies of consumer involvement versus no consumer involvement or of different ways of involving consumers [40]. We identified systematic reviews dealing with indirect evidence focusing on consumer involvement, for example in research agenda setting [31]. One review focused on involving patients in the planning and development of health care and another systematic review addressed which methods should be used to include the views of the public in policy documents [6,41]. We also found a review summarized values obtained by rating scale, time-trade off and standard gamble for different disease states [42]. In addition, we identified several articles that addressed whether patient values should be integrated in clinical practice guidelines [43].

### Whose values should WHO use when making recommendations?

Clinicians' values for health states differ from those of patients and among different clinician groups [14,44]. For example, the values physicians assign to stroke as an outcome and to adverse consequences (e.g., gastrointestinal bleeding) of treatment to prevent stroke in patients with atrial fibrillation differ from those of patients [14]. The values used in making recommendations should reflect those of the people affected. While there is widespread belief that values for health states also differ between patients and the general public, this belief is not supported by the available evidence. A systematic review of 33 studies found that preferences for hypothetical health states did not differ between patients and the public [39].

Since guidelines, for the most part, affect the use of limited public resources and, therefore, inevitably affect the general public, WHO guidelines should consider societal values and recognise when there may be important, legitimate differences in values across different cultures and settings. Judgements should be explicit and should be informed by input from those affected (including citizens, patients, clinicians and policy makers). Representation of all potentially relevant societies in groups developing global recommendations is not feasible. If differences in values are likely to affect a decision, such that people in different setting would likely make different choices about interventions or actions based on differences in their values, global recommendations should be explicit in terms of which values were applied and allow for adaptation after incorporating local values. It may also be possible to include consumers or other stakeholders in panels with the primary responsibility of questioning assumptions that are made about values, rather than representing the values of any particular group.

We found discussions of ethical considerations in guidelines and health technology assessments [2-4], but we did not find a systematic review of processes for addressing ethical considerations systematically and transparently. There is no standard way for doing this, although Hofmann has taken a step towards developing a practical approach by identifying relevant questions that could be asked in the context of health technology assessments [4].

### How should WHO ensure that appropriate values are integrated in recommendations?

The evidentiary basis for appropriate identification and representation of values in guideline development is limited. We did not identify a systematic review focusing on different ways of including values in clinical practice guidelines. A review by Ryan and colleagues focused on public preferences for healthcare [6]. The authors concluded that there was no single, best method to gain public opinion. The method must be carefully chosen and rigorously carried out in order to accommodate the question being asked. They recommend conjoint-based methods (including ranking, rating and choice-based), willingness to pay, standard gamble and time trade-off as quantitative techniques and one-to-one interviews, focus groups, Delphi technique and citizens' juries as qualitative techniques. There were only a few studies that conducted direct comparisons of methods at that time (up to 2000) and the review requires updating.

While the evidence about which method should be adopted is inconclusive, all of the methods mentioned above are acceptable. Methods that transform results of generic health related quality of life instruments into value scores can also be used but they have the disadvantage of requiring calculations and are based on mathematical assumptions in developing transformation equations. If data from primary research on societal or patient values are used, guideline panels should make explicit how these values were measured (e.g., they should specify whether a visual analogue scale, standard gamble or other methods were used).

Decision analysis and economic analyses are approaches to explicitly integrating values into guidelines, which may sometimes be useful [45]. Less formal methods include representation on guideline panels and consultation with consumers to inform judgements about the relative importance of the benefits and downsides of interventions based on rating scales. WHO guideline groups should uniformly apply explicit, transparent and clearly described methods for integrating values.

### How should users and consumers be involved in generating recommendations?

Consumers should be part of guideline groups, be able to contribute and be heard. Van Wersch and Eccles evaluated a guideline development program [46]. They described experience with three alternative methods of consumer involvement in guideline development: a) incorporating individual patients; b) a one off meeting with patients; and c) incorporating a consumer advocate in the guideline development group. They concluded that consumers should be involved in all stages of guideline development, while acknowledging that this is not straightforward, that there is no right way to accomplish this, and that more research is required to optimize the process and outcomes.

Indirect evidence about the involvement of consumers comes from studies evaluating the involvement of consumers in research priority setting. Oliver and colleagues systematically reviewed different methods of consumer involvement in research priority setting [31]. They concluded that "what we know about the advantages and disadvantages of methods for involving consumers in agenda setting rests on weak short-term evidence and almost entirely speculative long-term evidence". Telford and colleagues used a Delphi approach to identify principles and indicators of successful involvement of consumers in research [47]. They identified eight principles for the successful involvement in research that could also be used by WHO to ensure that consumers are adequately represented in guideline development projects (Table 1).

**Table 1:** The principles and indicators of successful consumer involvement in NHS research (from Telford et al.) [37]

Principle	Indicator(s)
1 The roles of consumers are agreed between the researchers and consumers involved in the research	• The roles of consumers in the research were documented
2 Researchers budget appropriately for the costs of consumer involvement in research	• Researchers applied for funding to involve consumers in the research • Consumers were reimbursed for their travel costs • Consumers were reimbursed for their indirect costs (e.g. carer costs)
3 Researchers respect the differing skills, knowledge and experience of consumers	• The contribution of consumers-skills, knowledge and experience were included in research reports and papers
4 Consumers are offered training and personal support, to enable them to be involved in research	• Consumers – training needs related to their involvement in the research were agreed between consumers and researchers • Consumers had access to training to facilitate their involvement in the research • Mentors were available to provide personal and technical support to consumers
5 Researchers ensure that they have the necessary skills to involve consumers in the research process	• Researchers ensured that their own training needs were met • in relation to involving consumers in the research
6 Consumers are involved in decisions about how participants are both recruited and kept informed about the progress of the research	• Consumers gave advice to researchers on how to recruit participants to the research • Consumers gave advice to researchers on how to keep participants informed about the progress of the research
7 Consumer involvement is described in research reports	• The involvement of consumers in the research reports and publications was acknowledged • Details were given in the research reports and publications • of how consumers were involved in the research process
8 Research findings are available to consumers, in formats and in language they can easily understand	• Research findings were disseminated to consumers involved in the research in appropriate formats (e.g. large print, translations, audio, Braille) • The distribution of the research findings to relevant • consumer groups was in appropriate formats and easily understandable language • Consumers involved in the research gave their advice on the choice of methods used to distribute the research findings

Any involvement of consumers requires a clear understanding of the evidence by consumers. Difficulties with medical terminology or other jargon are an important barrier to involvement. Well-informed and experienced consumers are more likely to interact with the guideline developers than those who are less informed or less familiar with medical terminology or other jargon that is used.

A large number of studies have been conducted using a variety of methods to elicit values for direct patient care questions, but this literature is not well summarised and it may be beyond the capacity of most groups developing recommendations to systematically review the literature relevant to the specific questions that they are addressing. The review by Morimoto and Fukui is limited to direct preference instruments and does not provide values for the full spectrum of diseases for which WHO develops recommendations [42]. However, this and similar reviews can provide some guidance because direct elicitation of values from a representative sample of people is rarely feasible.

WHO should consider developing a database or collaborating with others to establish a database of evidence about the relative values of common health outcomes across different cultures and settings, which could be used to inform the judgements made by groups making recommendations.

### How should values be presented in recommendations?

Values should always be considered in making recommendations, although they do not always influence the strength of a recommendation when they are uniformly shared among patients and society. While values should always be made explicit, WHO could restrict its presentation and labelling of values to those that are most important for decision-making. These recommendations could be flagged as being strongly influenced by values and include a presentation of whose values they represent. In particular recommendations should:

• Include a detailed description of how decisions were made about the relative importance of the consequences (benefits and downsides) of a decision. This should routinely be included in the methods section of a guideline.

• Values that influence recommendations should be reported along with the research evidence underlying the recommendations.

• When differences in values would lead to different decisions or there is important uncertainty about values that are critical to a decision, this should be flagged and reflected in the strength of the recommendation.

• Adaptable guideline templates that allow for integration of different values should be developed and used when differences in values are likely to be critical to a decision [48].

## Discussion

There is no high quality research informing the choice of whose values guideline panels should use or methods of consumer involvement. NICE has set examples and made advancements in involving consumers in guideline development. Feedback from consumers involved in the NICE process indicates that they value their involvement highly [28].

Oliver and colleagues identified a number of studies that evaluated different ways of involving consumers in research priority setting [31]. While the results of their systematic review are informative, there are two important limitations: 1) they focused on research priority setting and 2) direct comparison of different methods of consumer involvement were not found. A systematic review of comparative studies of methods for involving consumers in developing health care policy, research, clinical practice guidelines and patient information found five randomised trials, but none of these were relevant to informing decisions about how best to involve consumers in developing health care recommendations [40].

## Further work

We have identified a number of unresolved questions that require systematic reviews and additional research. A systematic review to evaluate the differences in values between consumers/patients and clinicians or experts is needed. Experimental work is needed that compares different strategies of consumer involvement in guideline development to evaluate whether more resource intensive approaches that include detailed methods to elicit and include values lead to different recommendations or other important differences.

A database of values assigned to specific health states may also facilitate the development of guidelines. Such a database should include information on the methods used and these methods should be explicitly stated when values are included in recommendations. While additional research on acceptable methods for eliciting values for inclusion in guidelines is required, one barrier is the philosophical and personal investment of researchers in particular methods. None of these available methods has demonstrated its superiority to others. Thus, achieving consensus on current best practice, that could be modified when new evidence become available, might be helpful.

Development of an appropriate checklist of questions that address key ethical considerations would help to ensure that these were addressed more systematically and facilitate reporting of important considerations, so that these were made more transparent. Adaptable guideline templates that allow for integration of different values should be developed and used when differences in values are likely to be critical to a decision [49]. Finally, better ways of communicating value-sensitive information need to be investigated.

## Competing interests

ADO and AF work for the Norwegian Knowledge Centre forthe Health Services, an agency funded by the Norwegian government that produces systematic reviews and health technology assessments. All three authors are contributors to the Cochrane Collaboration. ADO and HJS are members of the GRADE Working Group. HJS is documents editor and chair of the documents development and implementation committee for the American Thoracic Society and senior editor of the American College of Chest Physicians' Antithrombotic and Thrombolytic Therapy Guidelines.

## Authors' contributions

HJS prepared the first draft of this review. AF and ADO contributed to drafting and revising it. All authors read and approved the final manuscript.

## References

[B1] Torrance GW (1989). Utilities and quality-adjusted life years. Int J Tech Ass Health Care.

[B2] Cohen J (2004). Are clinical practice guidelines impartial. Int J Technol Health Care.

[B3] Giacomini MK (2000). Using practice guidelines to allocate medical technologies: an ethics framework. Int J Technol Health Care.

[B4] Hofmann B (2005). Toward a procedure for integrating moral issues in health technology assessment. Int J Technol Health Care.

[B5] Bennett KJ, Spilker B (1996). Measuring health state preferences and utilities: rating scale, time trade-off, and standard gamble techniques. Quality of life and pharmacoeconomics in clinical trials.

[B6] Ryan M, Scott DA, Reeves C, Bate A, van Teijlingen ER, Russell EM, Napper M, Robb CM (2001). Eliciting public preferences for healthcare: a systematic review of techniques. Health Technol Assess.

[B7] Froberg DG, Kane RL (1989). Methodology for measuring health-state preferences--I: Measurement strategies. Journal of Clinical Epidemiology.

[B8] Brazier J (1998). Deriving a preference-based single index from the UK SF-36 Health Survey. J Clin Epidemiol.

[B9] Torrance GW (1995). Multiattribute preferences functions: Health Utilities Index. Pharmacoeconomics.

[B10] Kaplan RM (1976). Health Status: Types of Validity and the Index of Well-being. Health Services Res.

[B11] EuroQol-Group (1990). EuroQol - a new facility for the measurement of health-related quality of life. Health Pol.

[B12] Guyatt G (2006). Grading strength of recommendations and quality of evidence in clinical guidelines. Chest.

[B13] Buller HRA (2004). Antithrombotic Therapy for Venous Thromboembolic Disease. CHEST.

[B14] Devereaux PJ, Anderson DR, Gardner MJ, Putnam W, Flowerdew GJ, Brownell BF, Nagpal S, Cox JL (2001). Differences between perspectives of physicians and patients on anticoagulation in patients with atrial fibrillation: observational study. Bmj.

[B15] Pritchard RS, Anthony SP (1996). Chemotherapy plus Radiotherapy Compared with Radiotherapy Alone in the Treatment of Locally Advanced, Unresectable, Non-Small-Cell Lung Cancer: A Meta-Analysis. Ann Intern Med.

[B16] Robinson LA, Wagner H, Ruckdeschel JC (2003). Treatment of Stage IIIA Non-small Cell Lung Cancer. Chest.

[B17] (2002). Methodology of guideline development. Neurosurgery.

[B18] AGREE, Collaboration (2003). Development and validation of an international appraisal instrument for assessing the quality of clinical practice guidelines: the AGREE project. Qual Saf Health Care.

[B19] Hayward RS WMC (1993). More informative abstracts of articles describing clinical practice guidelines. Ann Intern Med.

[B20] Rawlins MD, Culyer AJ (2004). National Institute for Clinical Excellence and its value judgments. Bmj.

[B21] Fretheim A (2006). Improving the Use of Research Evidence in Guideline Development: 3. Group composition. Health Res Policy Syst.

[B22] Oxman AD (2006). Improving the Use of Research Evidence in Guideline Development: 12. Incorporating considerations of equity. Health Res Policy Syst.

[B23] Schünemann HJ (2006). Improving the Use of Research Evidence in Guideline Development: 6. Determining which outcomes are important. Health Res Policy Syst.

[B24] (2003). Global Programme on Evidence for Health Policy. Guidelines for WHO Guidelines. EIP/GPE/EQC/2003.1.

[B25] The 2000 consensus conference on clinical practice guidelines. Toronto. Health Policy Forum; 2000 Nov 2.

[B26] Moynihan R (2005). A survey of producers of clinical practice guidelines and health technology assessments.

[B27] Burgers JS, Grol R, Klazinga NS, Makela M, Zaat J (2003). Towards evidence-based clinical practice: an international survey of 18 clinical guideline programs. Int J Qual Health Care.

[B28] Kelson MC (2005). The NICE Patient Involvement Unit. Evidence-Based Healthcare & Public Health.

[B29] Royle J, Oliver S (2004). Consumer involvement in the health technology assessment program. Int J Technol Assess Health Care.

[B30] Oliver S (1996). The progress of lay involvement in the NHS Research and Development Programme. J Eval Clin Pract.

[B31] Oliver S, Clarke-Jones L, Rees R, Milne R, Buchanan P, Gabbay J, Gyte G, Oakley A, Stein K (2004). Involving consumers in research and development agenda setting for the NHS: developing an evidence-based approach. Health Technol Assess.

[B32] Oliver S, Milne R, Bradburn J, Buchanan P, Kerridge L, Walley T, Gabbay J (2001). Involving consumers in a needs-led research programme: a pilot project. Health Expect.

[B33] Brainin M, Barnes M, Baron JC, Gilhus NE, Hughes R, Selmaj K, Waldemar G (2004). Guidance for the preparation of neurological management guidelines by EFNS scientific task forces - revised recommendations 2004. European Journal of Neurology.

[B34] Kelson MC (1999). Consumer collaboration, patient-defined outcomes and the preparation of Cochrane Reviews. Health Expectations.

[B35] CCNET Consumers in Cochrane. http://www.cochrane.org/consumers/homepage.htm.

[B36] Oxman AD (2006). Improving the Use of Research Evidence in Guideline Development: Introduction. Health Res Policy Syst.

[B37] Agency for Healthcare Research and Quality. http://www.guidelines.gov.

[B38] Guidelines International Network. http://www.g-i-n.net.

[B39] Dolders MGT, Zeegers MPA, Groot W, Ament A A Meta-analysis demonstrates no significant differences between Patient and Population Preferences. Journal of Clinical Epidemiology.

[B40] Nilsen ES Interventions for promoting consumer involvement in developing healthcare policy and research, clinical practice guidelines and patient information material. Cochrane Library.

[B41] Crawford MJ, Rutter D, Manley C, Weaver T, Bhui K, Fulop N, Tyrer P (2002). Systematic review of involving patients in the planning and development of health care. Bmj.

[B42] Morimoto T, Fukui T (2002). Utilities measured by rating scale, time trade-off, and standard gamble: review and reference for health care professionals. J Epidemiol.

[B43] Owens DK (1998). Spine update. Patient preferences and the development of practice guidelines. Spine.

[B44] Slomka J, Hoffman-Hogg L, Mion LC, Bair N, Bobek MB, Arroliga AC (2000). Influence of clinicians' values and perceptions on use of clinical practice guidelines for sedation and neuromuscular blockade in patients receiving mechanical ventilation. Am J Crit Care.

[B45] Guyatt G, Guyatt G RD (2002). Moving from Evidence to Action. Incorporating patient values. Users' Guide to the Medical Literature: A Manual for Evidence-Based Clinical Practice.

[B46] van Wersch A, Eccles M (2001). Involvement of consumers in the development of evidence based clinical guidelines: practical experiences from the North of England evidence based guideline development programme. Qual Saf Health Care.

[B47] Telford R, Boote JD, Cooper CL (2004). What does it mean to involve consumers successfully in NHS research? A consensus study. Health Expect.

[B48] Schünemann HJ, Fretheim A, Oxman AD Templates, adaptation and the role of international guidelines.

[B49] Schünemann HJ (2006). Improving the Use of Research Evidence in Guideline Development: 13. Adaptation, applicability and transferability. Health Res Policy Syst.

